# Experimental Assessment of Traction Force and Associated Fetal Brain Deformation in Vacuum-Assisted Delivery

**DOI:** 10.1007/s10439-024-03665-z

**Published:** 2024-12-22

**Authors:** Estelle Pitti, Lotta Herling, Xiaogai Li, Gunilla Ajne, Matilda Larsson

**Affiliations:** 1 Biomedical Engineering and Health Systems, KTH Royal Institute of Technology, Stockholm, Sweden; 2 Clinical Science Intervention and Technology-CLINTEC, Karolinska Institutet, Stockholm, Sweden; 3 Pregnancy Care & Delivery, Karolinska University Hospital, Stockholm, Sweden

**Keywords:** Vacuum-assisted delivery, Fetal head phantom, Experimental setup, Traction force, Brain deformation

## Abstract

Vacuum-assisted delivery (VAD) uses a vacuum cup on the fetal scalp to apply traction during uterine contractions, assisting complicated vaginal deliveries. Despite its widespread use, VAD presents a higher risk of neonatal morbidity compared to natural vaginal delivery and biomechanical evidence for safe VAD traction forces is still limited. The aim of this study is to develop and assess the feasibility of an experimental VAD testing setup, and investigate the impact of traction forces on fetal brain deformation. A patient-specific fetal head phantom was developed and subjected to experimental VAD in two testing setups: one with manual and one with automatic force application. The skull phantom was 3D printed using multi-material Polyjet technology. The brain phantom was cast in a 3D-printed mold using a composite hydrogel, and sonomicrometry crystals were used to estimate the brain deformation in three brain regions. The experimental VADs on the fetal head phantom allowed for quantifying brain strain with traction forces up to 112 N. Consistent brain crystal movements aligned with the traction force demonstrated the feasibility of the setup. The estimated brain deformations reached up to 4% and correlated significantly with traction force (*p* < 0.05) in regions close to the suction cup. Despite limitations such as the absence of scalp modeling and a simplified strain computation, this study provides a baseline for numerical studies and supports further research to optimize the safety of VAD procedures and develop VAD training platforms.

## Introduction

Although childbirth is a physiological process that often occurs naturally, complications can arise impeding spontaneous vaginal delivery [1]. To follow the recommendation of the World Health Organization to avoid cesarean delivery when it is not necessary [2, 3], a key option is to provide additional assistance (force and direction) to the parturient woman through instrumental vaginal delivery (IVD) [1, 4, 5]. IVD consists of two families of techniques [5]: obstetric forceps delivery and vacuum-assisted delivery (VAD), which together account for 10–15% of all vaginal deliveries in Europe, Canada, and Australia/New Zealand [6–8]. VAD was introduced later than obstetric forceps in an effort to reduce maternal trauma [4, 9]. It is now the most common type of IVD, accounting for 0.5–16.4% of all deliveries in Europe [6], with a prevalence of around 5% in Sweden [10].

Indications for clinical use of VAD are provided by national [11] and international guidelines [12, 13], which recommend VAD when complications arise during the second stage of labor, such as fetal or maternal distress, intrapartum infection, maternal exhaustion, poor contractions, or prolonged labor [4, 5]. Since the development of the device by Dr. Tage Malmström in 1953 [9] and its improvement by Dr. Geoffrey Bird in the 1960s [14], the device design has remained largely unchanged [4]. It consists of a vacuum cup placed on the fetal scalp sealed by negative pressure. During the mother’s contraction, traction force is applied to the handle of the system to assist the delivery of the fetus [1, 4, 5].

Despite its common use, VAD presents a higher risk of neonatal morbidity compared to vaginal delivery [15]. Short-term fetal complications are well known as they are directly observed clinically. The most common fetal complications after VAD, such as caput succedaneum and cephalohaematomas, are in most cases not severe and may resolve on their own after a few weeks [16]. However, more severe short-term fetal complications may occur following VAD such as a subgaleal hematoma (0.4–2.5% incidence) [15, 17], intracranial hemorrhage (ICH) including intraventricular hemorrhage (0.1–0.8% symptomatic ICH incidence) [15, 18], seizures, asphyxia, scalp, and cranial fractures [19]. Besides the short-term complications, neonatal morbidity may lead to neurodevelopmental problems during childhood [20]. Preliminary data on the long-term complications associated with VAD indicate that VAD increases the risk of motor skill and perception problems, and attention-deficit/hyperactivity disorders in children later in life compared to spontaneous vaginal delivery [21].

Neonatal adverse outcomes may result from various injury mechanisms, such as cup misplacement [22], cup detachment [4, 22, 23], or other suction cup characteristics (including manufacturer, material, and dimensions) [24]. These can lead to scalp and subcutaneous tissue injuries [15, 17, 18], as well as reduced brain oxygenation caused by altered blood perfusion in the fetal head, resulting from changes in intracranial pressure due to the applied traction force [21, 25]. Recent interest in the level of traction force during VAD has shown that the short- and long-term adverse outcomes may be due to high traction force. As such, the risk of admission to the neonatal intensive care unit is three times higher with a high traction force [23]. In addition, studies have shown that high VAD traction force increases the risk of severe neonatal outcome and that limiting the force to 343 N min (area under the curve of the traction force over time) would reduce the incidence of a severe neonatal outcome by 86% [26]. The safe limit of traction force in VAD has been a topic of interest over time, starting with Malmström, who determined that the maximum force that can be applied to the cup before detachment is 220 N, given a high negative pressure inside the cup (−80 kPa) and the largest diameter of the cup (6 cm) [4, 9]. Other studies have shown that a traction force of approximately 120 N is sufficient to guarantee the success of the VAD procedure in 80% of cases [4, 27, 28]. These studies claim that the safety of the traction force is assured by the suction cup physics, i.e., that unintentional cup detachment occurs if the traction force exceeds the maximum force of the suction cup defined by the negative pressure inside the cup and its diameter [4, 28]. However, later studies found that 34% of VAD deliveries reached a traction force higher than 216 N and that a traction force up to 400 N could be observed without cup detachment [29].

Current studies on safe levels of VAD traction force use outdated and oversimplified fetal head models, neglecting the anatomy, geometry, and mechanical properties of the fetal head. For example, Malmström used a rubber balloon [9], and Duchon et al. [30] employed a 10-cm solid sphere covered with canine hindquarters to study various aspects of VAD traction. Training in obstetrics also employs fetal head phantoms to improve VAD procedures. However, most commercially available models and advanced simulators like BirthSim software focus on anatomical representation and overlook the unique mechanical properties and structures of the skull bones and fontanelles, and do not include a fetal brain model [31, 32].

What emerges from the literature is the lack of knowledge to define safe application of VAD traction force. Even if early studies investigated the safe limit of traction force in VAD, particularly in relation to cup detachment events [22] and clinical perspectives [9, 23, 27, 28, 33], detailed studies on the biomechanics of the fetal head in response to applied traction force is lacking [4]. In addition, the lack of force-level guidelines and force-related safety training are evident since the assessment of traction force during VAD is not part of the clinical routine, leading clinicians to rely on subjective judgment, which raises safety concerns as the level of traction force is frequently underestimated [4, 29]. Moreover, the causes of the observed adverse outcomes in VAD are not known [33–35]. There is still a debate whether the increased risk of neonatal morbidity and especially long-term complications in VAD are due to clinical biases (e.g., adverse clinical conditions before childbirth, during delivery, and anatomical aspects of the fetal head or the mother’s birth canal) or to inherent aspects of the VAD procedure (e.g., amplitude and rate of traction force) [4, 33]. Furthermore, the literature also highlights the lack of appropriate physical models to study the effect of VAD traction force on the risk of fetal brain injury [4]. The practical and ethical difficulties of in vivo and post-mortem human subject (PMHS) testing on the fetal brain emphasize the need for physical models providing reference data to validate finite element models (FEM), similar to the field of head impacts [36–38].

Therefore, this study aimed to develop an experimental VAD testing setup including a fetal head phantom with realistic geometry and mechanical properties, to investigate the feasibility of the VAD testing setup, and to study the impact of the traction force on brain deformation during experimental VAD. First, a fetal head phantom was developed to replicate the key anatomical features and mechanical properties of a human fetal head. Two experimental VAD testing setups were then implemented, applying traction force either manually or automatically. Both setups simulated the onset phase of clinical traction force, allowing for the study of the brain deformation caused by peak traction forces. This research is a step toward further investigations into safe traction force limits, safety-related training for obstetricians, and numerical simulations of VAD.

## Methods

The impact of the traction force on fetal brain deformation was investigated in a physical fetal head phantom in two experimental VAD testing setups. One setup was designed for the translation into clinical situations and training in obstetrics with the application of manual traction force and an external force gage for force measurement. The other setup was designed to test VAD in a controlled situation using a mechanical testing machine to apply and measure the traction force. The deformation of the brain was assessed using sonomicrometry.

### Fetal Head Phantom Development

A fetal head phantom, including a skull and a brain phantom, was constructed based on segmentation of CT scan images of a newborn and 3D-printing technology. CT scan images (image resolution of 0.26 * 0.26 * 0.5 mm^3^) of the head of 207 newborn PMHS were requested from the open-source New Mexico Decedent Image Database (NMDID) [39]. One newborn dataset was selected based on visual assessment of head geometry by two obstetricians, and the NMDID metadata [below 1 month of age, weight between 2.5 and 5 kg, excluding causes of death related to brain damage (e.g., cerebrovascular accident, congenital defects, and meningitis), and excluding causes of death preventing VAD (e.g., stillbirth and prematurity)]. Another major criterion for selecting the newborn dataset was the 3D-printing suitability of the fetal head geometry as the chosen geometry needed to exhibit deformation from the head molding process within the limits of what could be accurately produced by 3D printing. The geometry of the skull bones, the sutures and fontanelles, and the brain were extracted by manually segmenting the CT scan images using Slicer 3D v.5.0.3. First, the skull bones were segmented. Then, the space between the skull bones was selected to obtain a separate segmentation of sutures and fontanelles. Subsequently, the brain segmentation was extracted by adjusting the image contrast. The thickness of the segmented skull was measured across different locations, with an average thickness of approximately 1.6 mm.

**Fig. 1:**
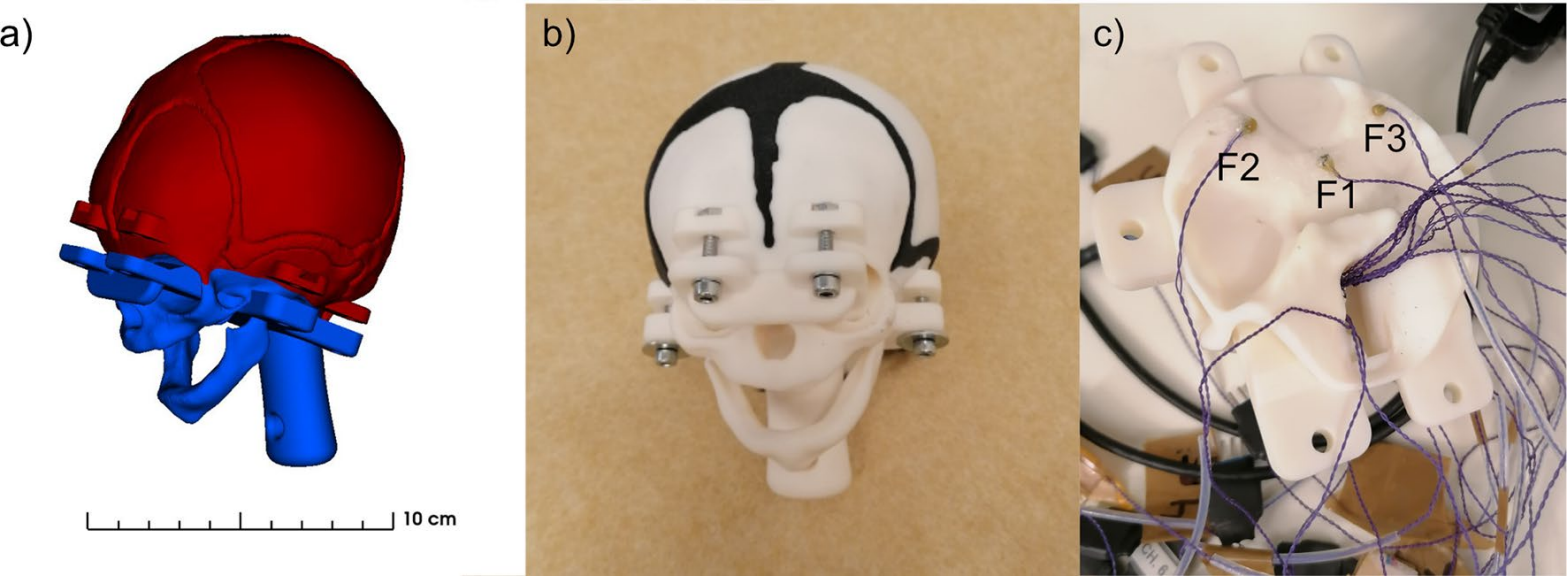
Skull phantom. **a** Lower part (blue) and upper part (red) of the CAD skull model, **b** lower and upper part of the 3D-printed skull phantom mounted by a screwing system, and **c** lower part of the skull phantom with the three fixed piezoelectric crystals (F1, F2, F3)

The segmentation of the skull bones, the sutures, and fontanelles was combined in a computer-aided design (CAD) model structured in two parts: separated below the squamous sutures and above the eyes (Fig. 1a). A screwing system was added to the skull phantom to provide a reproducible way of inserting the brain phantom and ensuring the structural integrity of the skull phantom during the experiments (Fig. 1b). The separation and screw locations were carefully chosen to minimize the impact on critical skull regions, particularly in the context of VAD. The separation was not made along any sutures or fontanelles; instead, it was positioned below all sutures and fontanelles and beneath the occipital, frontal, and parietal bones, while maximizing the size of the constricted opening to insert the brain phantom inside the skull cavity. The geometry of the neck bone was simplified to enable the fixation of the fetal head phantom on the bottom of a water tank, as explained in "Experimental VAD Testing Setups" Section. The two parts of the skull phantom, shown in Fig. 1a, were printed separately using multi-material Polyjet technology (Stratasys, Rehovot, Israel) [40]. This technology enabled the upper part of the phantom to be printed as a single piece including the skull bones, the sutures, and the fontanelles with different Stratasys materials (Stratasys, Rehovot, Israel) based on previous work by Jones et al. [41, 42] to best represent the mechanical properties of the biological equivalent as presented in Table 1 (data extracted from [42]). Three piezoelectric crystals of 2 mm diameter (Sonometrics, London, Canada) were fixed with silicon in 1 mm deep cavities (referred to as the fixed crystals; F1, F2, F3) inside of the lower part of the skull phantom (Fig. 1c) to assess the deformation of the brain using sonomicrometry and trilateration, further detailed in "Brain Phantom Deformation Assessment" Section.

**Table 1:** Comparison of mechanical properties between biological components of the fetal skull and selected materials for the fetal skull phantom (*E*_1_ parallel to the fibers, *E*_2_ perpendicular to the fibers,* C*_1_ and *C*_2_ two parameters of the hyperelastic Mooney–Rivlin model) [42–50]

	Skull phantom				
	Lower part	Upper part			
Biological component	Facial bones and simplified neck bones	Parietal skull bones	Frontal skull bone	Occipital skull bone	Sutures and fontanelles
*Young’s modulus (MPa)*					
Peterson (2003)	9700–21,200				
McElhaney (1970)	4460				
McPherson (1980)		*E*_1_: 3880	*E*_1_: 3057		
		*E*_2_: 951	*E*_2_: 1700		
Collier (2024)				*E*_1_: 2839	
				*E*_2_: 472	
Coats (2006)				29–551	3.8–6.4
Galford (1970)					31.5
Bylski (1986)					*C*_1_: 1.2
					*C*_2_: 0.3
*Tensile strength (MPa)*					
Coats (2006)		8.4–53.7		3.1–18.5	2.2–7.2

Phantom material	VeroWhite Plus RGD835			RigidLightGrey RGD 8510-DM	FLX-9870-DM
*Young’s modulus (MPa)*					
Ghaidaa (2018)	1471–2201			1412–1962	4.3–8.6
*Tensile strength (MPa)*					
Ghaidaa (2018)	24–25.7			23.8–24.5	0.8–0.9

**Fig. 2:**
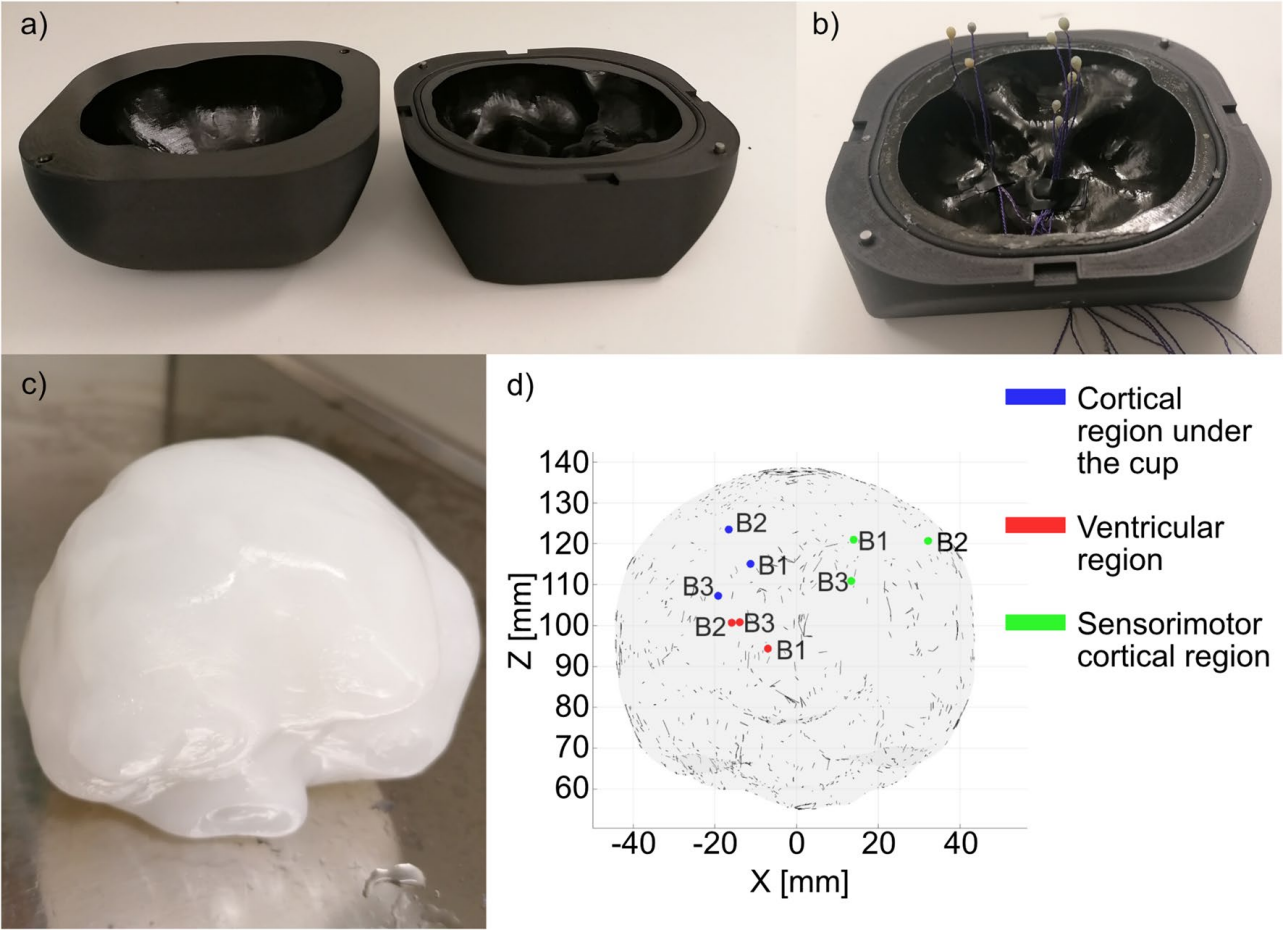
Brain phantom construction. **a** The two parts of the brain mold, **b** brain mold with internally positioned brain crystals, **c** brain phantom, and **d** schematic of the brain phantom with initial crystal positions in the *X*–*Z* plane of the initial reference frame, i.e. the frontal view

Based on the brain segmentation, a mold of the brain phantom was 3D printed in two parts (Fig. 2a) with an inlet at the bottom of the brain phantom, using the Onyx Pro^TM^ Markforged printer (Markforged, Waltham, Massachusetts, United States) and its compatible material Onyx (Markforged, Waltham, Massachusetts, United States). To assess brain deformation, three sets of three piezoelectric crystals were placed in different regions of the brain (the cortical region located under the cup, the ventricular region, and the sensorimotor cortical region). Those nine piezoelectric crystals in the brain phantom referred to as brain crystals (three crystals per brain region; B1, B2, B3). Compared to the three fixed crystals in the skull phantom (Fig. 1c), the brain crystals moved according to the deformation of the brain. The selection of brain regions was based on the neural regions of interest in the context of VAD and newborns’ brains [51, 52]. Placement of the brain crystals in the brain phantom was achieved by placing the crystal wires to the bottom of the brain mold before the brain material was cast (Fig. 2b).

The composite hydrogel (CH) mimicking the brain was composed of polyvinyl alcohol (PVA, 99.3–100.0% hydrolyzed, M.W. approx. 146,000–186,000, Thermo Scientific Chemicals, Waltham, Massachusetts, United States) and Phytagel (PHY, Gellan Gum Powder, Thermo Scientific Chemicals, Waltham, Massachusetts, United States). It was produced by separately dissolving 6% PVA and 0.85% PHY in water for an hour at 90 °C using a stirring hot plate. Subsequently, these solutions were combined in a 1:1 weight ratio at 70 °C for 30 min with continuous stirring. The resulting solution, while still hot, was poured into the brain mold, frozen at − 25 °C for 20 h, and then thawed at ambient temperature for 10 hours, completing one freezing–thawing cycle (Fig. 2c). The brain phantom was then stored in water. This hydrogel and preparation procedure was suggested by Forte et al. [53] because of its advantageous mechanical properties such as tunable stiffness and rate-dependent characteristics, as well as for its similarity to the biological brain in terms of compressive stress and hyperelastic behavior. The location of the brain crystals inside the brain phantom in three different brain regions after production of the CH poured in the brain mold can be found in Fig. 2d.

Subsequently, the brain phantom with the nine brain crystals was inserted in the skull phantom, and the two parts of the skull phantom were screwed together. Although the skull phantom was 3D printed in two halves, the brain phantom had to be slightly trimmed to fit through the opening of the cavity of the upper part of the skull phantom (Fig. 1a). The cuts of the brain phantom were made on one side of the phantom (0.5 cm at the largest thickness and 4 cm at the largest height) and at the bottom (1.5 cm at the largest thickness), corresponding to small volumes of white matter near the brainstem and temporal lobe in the human brain. The cut slices were then inserted in the skull phantom to preserve the overall mass and geometry of the brain phantom.

### Experimental VAD Testing Setups

**Fig. 3:**
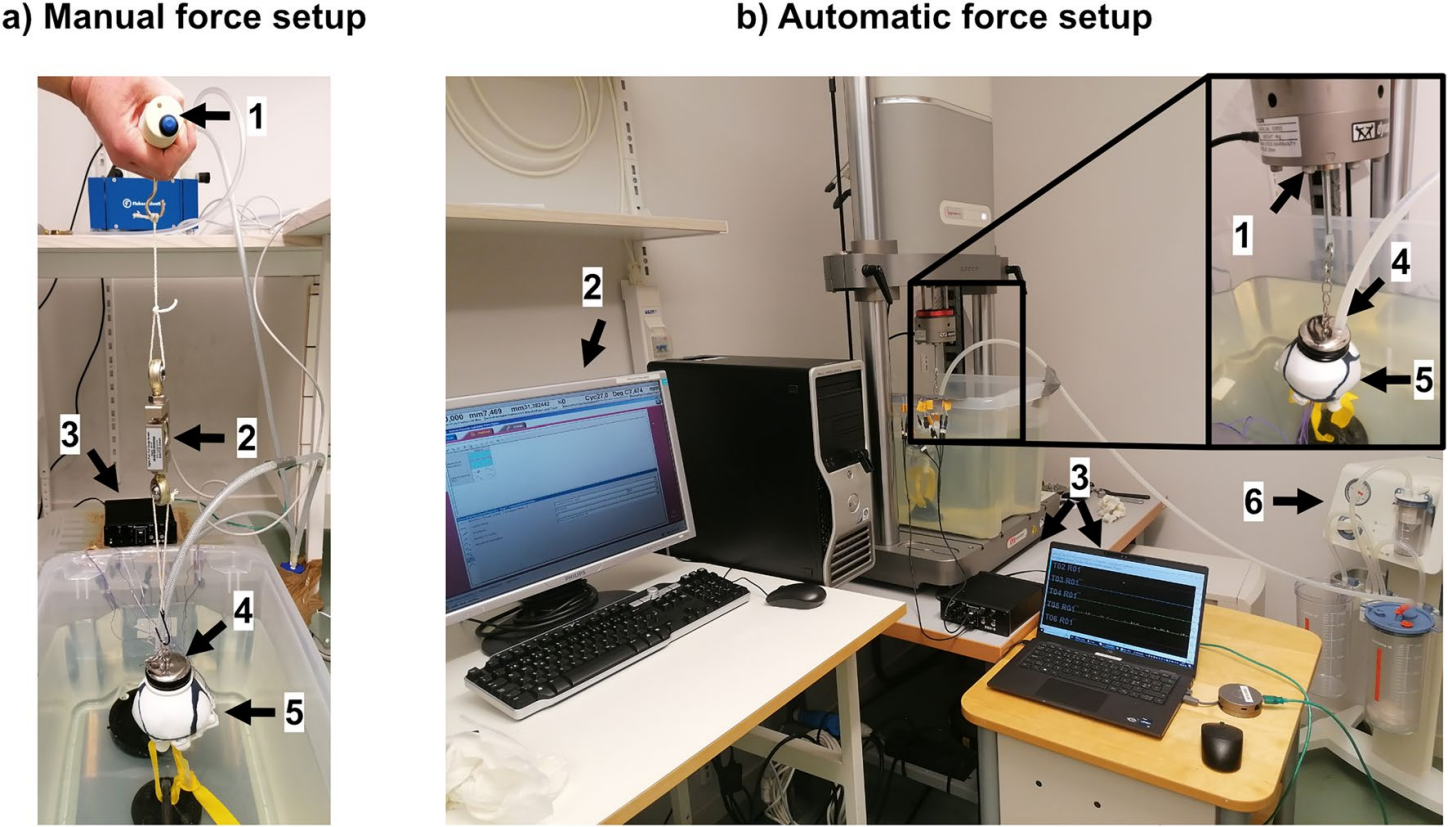
Experimental VAD testing setups, **a** manual force setup: 1 VAD handle, 2 force gage, 3 sonomicrometry system, 4 VAD cup, 5 fetal head phantom, and **b** automatic force setup: 1 Instron actuator, 2 Instron software, 3 sonomicrometry system, 4 VAD cup, 5 fetal head phantom, 6 vacuum pump

Two experimental VAD testing setups were built, a setup with manual application of traction force and an external force gage for force measurement (Fig. 3a), and a setup with automatic application and measurement of traction force using a mechanical testing machine (Instron V1.3, Norwood, Massachusetts, United States) with a 4-kg load cell of approximately 5000-N capacity and a calibrated maximum displacement error equal to 0.03% (Fig. 3b). In both setups, the fetal head phantom was attached at the bottom of a water tank by a rope connecting the fixed hook at the bottom of the tank and the simplified neck bones of the phantom. Water was inserted into the phantom to represent the cerebrospinal fluid [53] by shaking the fetal head phantom slightly until no air bubbles were released.

A clinical metal Bird cup (Medela Medical AB, Täby, Sweden) with a diameter of 5 cm was placed on the fetal head phantom at the optimal location for suction cup placement in clinical practice, the so-called flexion point [4]. The suction cup was kept out of the water and the seal between the fetal head, and the suction cup was ensured by a clinical vacuum pump (− 80 kPa, Dominant Flex, Medela Medical AB, Täby, Sweden). The fetal head phantom did not include any scalp model. Therefore, a 5-mm-thick cushion (ethylene propylene diene terpolymer) was glued around the edge of the suction cup to increase adhesion with the skull phantom.

This study replicates only the onset phase of a typical traction force curve used in clinical settings [26], reaching a peak of approximately 150 N at an application rate of 30 N/s. The focus was on examining the dynamics of the force–strain relationship by applying various amplitudes of traction forces at this consistent application rate. In the manual force setup (Fig. 3a), an operator manually applied traction force on the suction cup handle to replicate the application rate of clinically applied VAD traction forces [26], including human variability in the procedure. A digital force gage (FH 1K Sauter, Balingen, Germany) recorded the force applied between the suction cup and the handle with a precision of 0.5 N and a sampling rate of 10 Hz. The traction force was displayed in real time on the software (Sauter AFH-FAST FD Measuring Software Version 1.0.9.3, Balingen, Germany) and saved in an XML file that was then imported in MATLAB R2022b (Natick, Massachusetts, United States). In the automatic force setup (Fig. 3b), the mechanical testing machine was used to apply and record the traction force. A hook was mounted on the load cell of the machine from which the suction cup was attached. The experiment was conducted using a method of load control where the amplitude of traction force applied to the phantom was imported into the software in the form of a sampled data waveform. The waveforms were defined as a trapezoidal shape over a duration of 10 s, starting at 0 N the first 0.5 s and increasing until reaching a predefined peak with a rate of 30 N/s, keeping a plateau before decreasing with the same rate and reaching 0 N for the last 0.5 s. The force curves, recorded by the WaveMatrix (Instron Software version 1.3.214.0, Norwood, Massachusetts, United States) with a sampling rate of 1000 Hz, were saved in excel files and then imported in MATLAB R2022b (Natick, Massachusetts, United States).

For both experimental VAD testing setups, the peak traction force was increased in 20 N increments, starting at 30 N and up to 110 N. In the manual force setup, each experiment was repeated three times, one for each set of brain crystals because the sonomicrometry system used in this study was limited to six active channels (three fixed crystals and three brain crystals). In addition, the experiments of 70 N and 110 N peak force were repeated four times to perform repeatable measurements with two crystal sets; the cortical region under the cup and the ventricular region. In the automatic force setup, the cortical region under the cup was the only crystal set assessed and the 70 N force peak was repeated four times to assess repeatability. The experiments in the manual force setup were performed before using the automatic force setup.

### Brain Phantom Deformation Assessment

**Fig. 4:**
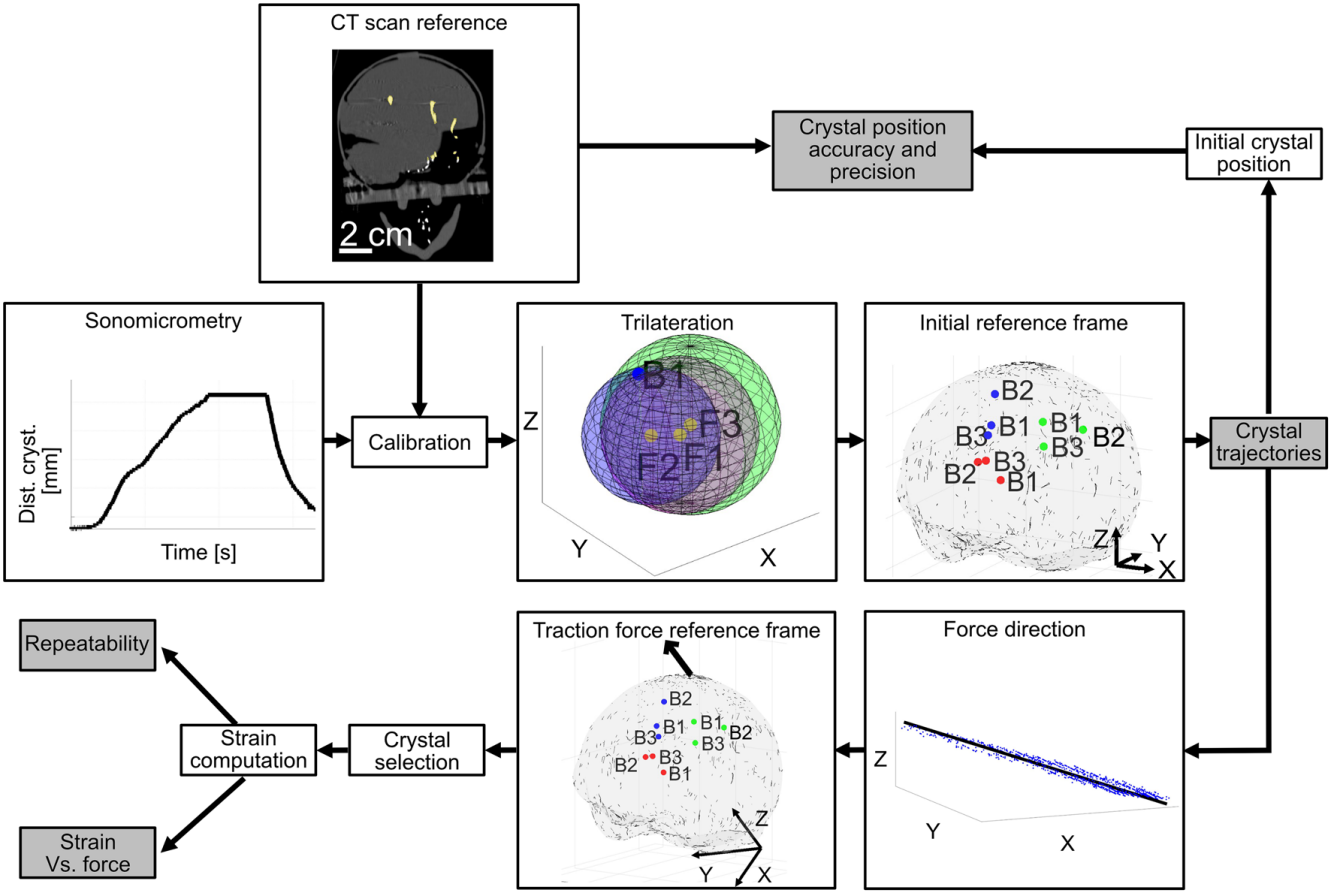
Post-processing workflow of brain phantom deformation with gray boxes referring to the four outputs of the method presented in the results section. In Trilateration: the three spheres (red, blue, and green) are defined by their centers (yellow dots) being the positions of the fixed crystals (F1, F2, F3) and the inter-crystal distances between each fixed crystal and a brain crystal (B1, blue dot). Initial reference frame/Traction force reference frame: initial position of the brain crystals in the cortical region under the cup (blue), the ventricular region (red), and the sensorimotor cortical region (green). Traction force reference frame: the black arrow designates the manual traction force direction

The deformation of the brain phantom was assessed using a sonomicrometry system (Sonometrics, London, Canada) by measuring inter-crystal distances of the piezoelectric crystals in the fetal head phantom (sampling rate of 200.3 Hz and transmit pulse of 406.25 ns). Brain strain was extracted following the post-processing steps summarized in Fig. 4. The first step involved filtering of the inter-crystal distances over time to eliminate outliers by removing values that were more than three times the scaled median absolute deviation (MAD) away from the median. Then, to calibrate the sonomicrometry system, the fetal head phantom was subjected to a CT scan (GE Discovery MI4 (PET/CT), GE HealthCare, Illinois, Chicago, United States) (Helical acquisition mode, spiral pitch factor of 0.53, bone convolution kernel, and image resolution 0.39 * 0.39 * 0.625 mm^3^). Segmentations of the skull phantom and the piezoelectric crystals were carried out using Slicer 3D v. 5.0.3 to extract the initial crystal positions. The positions of the crystals identified in the CT scan images served as the initial reference points for calibrating the sonomicrometry data. A calibration factor (*f*) (one for each pair of crystals), computed as the ratio of the inter-crystal distance by the CT scan images (${D}_{\text{CT}}$) and the inter-crystal distance by the sonomicrometry (${D}_{\text{sono}}$) in the initial time period ${(t}_{0})$ (from 0 to 0.25 s), was multiplied by the sonomicrometry data over the time of the experiment (*t*) as follows:

1 $$\text{Calibrated } {\text{D}}_{\text{sono}}\left(t\right)= f*{D}_{\text{sono}}\left(t\right)= \frac{{D}_{\text{CT}} }{{D}_{\text{sono}}\left({t}_{0}\right)}*{D}_{\text{sono}}\left(t\right).$$

The calibrated inter-crystal distances ($\text{Calibrated } {\text{D}}_{\text{sono}}(t)$) were fed as inputs in a trilateration algorithm. The algorithm estimated the 3D positions of each brain crystal (*X*, *Y*, *Z* coordinates) over time in the initial reference frame (Fig. 4) by calculating the intersection of three spheres. The three fixed crystal positions (considered constant throughout the experiment) were the sphere centers, and the radii were the calibrated inter-crystal distances between the fixed crystals and each brain crystal. The trilateration output was then filtered to remove outliers by removing values that were more than three times the scaled MAD away from the median.

The difference between the initial crystal positions computed by the trilateration algorithm and the initial crystal positions from the CT scan images used as reference positions, was calculated to evaluate the accuracy and precision of the crystal position tracking presented in "Crystal Position Accuracy and Precision" Section. The 3D coordinates of each brain crystal computed from the trilateration algorithm were projected onto the three Cartesian planes in the initial reference frame, and their trajectories were visualized by connecting the positions over time. A semi-automatic 2D linear regression was performed on each Cartesian plane (*XY* plane, *XZ* plane and *YZ* plane) to find the best-fitting line, minimizing the distance between the 3D coordinates of each brain crystal in each time step and the line. The angle between the line and the horizontal axis of each Cartesian plane, in degrees, was estimated for each brain crystal in every experiment to estimate the direction of the crystal trajectories in each Cartesian plane. The direction of the traction force applied in each VAD setup was determined as the average of crystal trajectories across all experiments and brain crystals. In the manual force setup, it was based on three crystal sets, while in the automatic force setup, it was based on one unique crystal set; the cortical region under the cup. The traction force direction was used to build the traction force reference frame with the *Z*-axis aligned with the traction force direction. The displacement of each crystal was then projected in the traction force direction (*Z*-axis of the traction force reference frame) before the application of a filter using Gaussian-weighted average over each window of 0.26 s.

For each crystal set, the crystal pair with the best alignment and the largest inter-crystal distance in the direction of the traction force was selected for further analysis (cortical region under the cup: crystals B2–B3, ventricular region: crystals B1–B3, and sensorimotor cortical region: crystals B1–B3) as explained in "Crystal Trajectories" Section. The inter-crystal displacement in the force direction for the selected crystal pairs was filtered by removing outliers that were more than three times the scaled MAD away from the median and Gaussian-weighted average over each window of 0.26 s. The strain was then calculated as follows:

2 $$\text{Strain} \left(t\right)= \frac{\left(D\left(t\right)- D\left({t}_{0}\right)\right)}{D\left({t}_{0}\right)},$$

where *D* represents the inter-crystal displacement in the force direction over the time period of the experiment (*t*) and over the initial time period $({t}_{0})$ (from 0 to 0.25 s). The strain was then filtered by convolving with a block of 1.04 s.

The strain peaks and the crystal displacement peaks were then aligned in time with the corresponding force peaks. Pearson correlation coefficient was calculated to examine the correlation between peak force and peak strain with a significance level of 0.05. The repeatability of the experiments was investigated by plotting the mean and the standard deviation of the force, crystal displacement, and strain profiles of repeated experiments over time after aligning the peak values in time.

## Results

A total of 39 experiments were performed on the fetal head phantom. Within the first 30 experiments in the manual force setup, three experiments were eliminated due to cup detachment occurring when traction forces reached approximately 110 N peak (111 N, 111.5 N, and 114.5 N). A traction force of around 105 N was, therefore, targeted as the maximum force peak for each crystal set, instead of the predefined force peak of 110 N. After the experiments in the manual force setup, nine experiments were conducted in the automatic force setup using the same fetal head phantom. During the third repetition of the experiment applying 110 N of traction force in the automatic force setup, the fetal head phantom fractured at the sagittal suture and at the junctions of the squamous sutures and skull bones on both sides. This prevented the completion of the planned experiments, which included four repetitions with a 110 N force peak and tests involving other brain regions, as done in the manual force setup. Since the damaged fetal head phantom could not be repaired or recreated, the experiments for this study were concluded.

### Crystal Position Accuracy and Precision

The crystal position errors, computed as the distance between initial crystal positions estimated by the trilateration algorithm and the references from the CT scan images, are reported in Table 2. The results show that the acquisition and processing (calibration and trilateration algorithm) of sonomicrometry data exhibit accuracy in locating brain crystals in space, with small crystal position errors across all crystal sets in both experimental VAD testing setups. In addition, the small standard deviations indicate consistent accuracy across experiments, demonstrating that the estimation of the crystal positions remains precise and unaffected by random errors.

**Table 2:** Crystal position errors (absolute distances) of the three brain crystals (B1, B2, B3) in the initial time period (from 0 to 0.25 s), computed as the distance between initial crystal positions estimated by the trilateration algorithm and the references from the CT images over the different experiments performed with each crystal set in the manual force setup; cortical region under the cup (*N* = 11), ventricular region (*N* = 11), and sensorimotor cortical region (*N* = 5), and for the crystal set of the cortical region under the cup in the automatic force setup (*N* = 9)

	Crystal position error: mean (SD, mm)		
Crystal	B1	B2	B3
Crystal set			
*Manual force setup*			
Cortical region under the cup	0.2295E−4 (0.0521E−4)	0.9431E−4 (0.2469E−4)	0.3864E−4 (0.1585E−4)
Ventricular region	0.3452E−3 (0.7053E−3)	0.2061E−3 (0.0534E−3)	0.0160E−3 (0.0027E−3)
Sensorimotor cortical region	0.0001 (0.0000)	0.0262 (0.0472)	0.0055 (0.0130)
*Automatic force setup*			
Cortical region under the cup	0.0001 (0.0000)	0.0154 (0.0170)	0.0003 (0.0010)

### Crystal Trajectories

Table 3 reports the direction of the crystal trajectories computed as the angle between the 2D linear regression line and the horizontal axis of each Cartesian plane in the initial reference frame (*XY* plane, *XZ* plane, and *YZ* plane) for each crystal set in both experimental VAD testing setups. The standard deviations of the direction of the crystal trajectories within each crystal set were consistently below 6.4° for the manual force setup and below 9.1° for the automatic force setup, indicating uniform movement of brain crystals in the estimated direction of the applied traction force.

**Table 3:** Crystal trajectory direction of the brain crystals (B1, B2, B3) projected in the Cartesian planes of the initial reference frame (*XY* plane, *XZ* plane, and *YZ* plane) over the different experiments performed with each crystal set in the manual force setup; cortical region under the cup (*N* = 11), ventricular region (*N* = 11), and sensorimotor cortical region (*N* = 5), all crystal sets in the manual force setup and for the crystal set of the cortical region under the cup in the automatic force setup (*N* = 9)

	Crystal trajectory direction: mean (SD, °)		
2D plane	*XY* plane	*XZ* plane	*YZ* plane
Crystal set			
*Manual force setup*			
Cortical region under the cup	64.14 (5.42)	− 69.49 (4.57)	− 52.93 (6.36)
Ventricular region	63.67 (6.12)	− 74.59 (4.34)	− 59.18 (5.24)
Sensorimotor cortical region	63.76 (3.83)	− 66.60 (2.53)	− 49.09 (4.54)
All crystal sets	63.85 (0.25)	− 70.23 (4.05)	− 53.73 (5.09)
*Automatic force setup*			
Cortical region under the cup	45.41 (8.83)	− 61.59 (6.18)	− 63.75 (9.11)

**Fig. 5:**
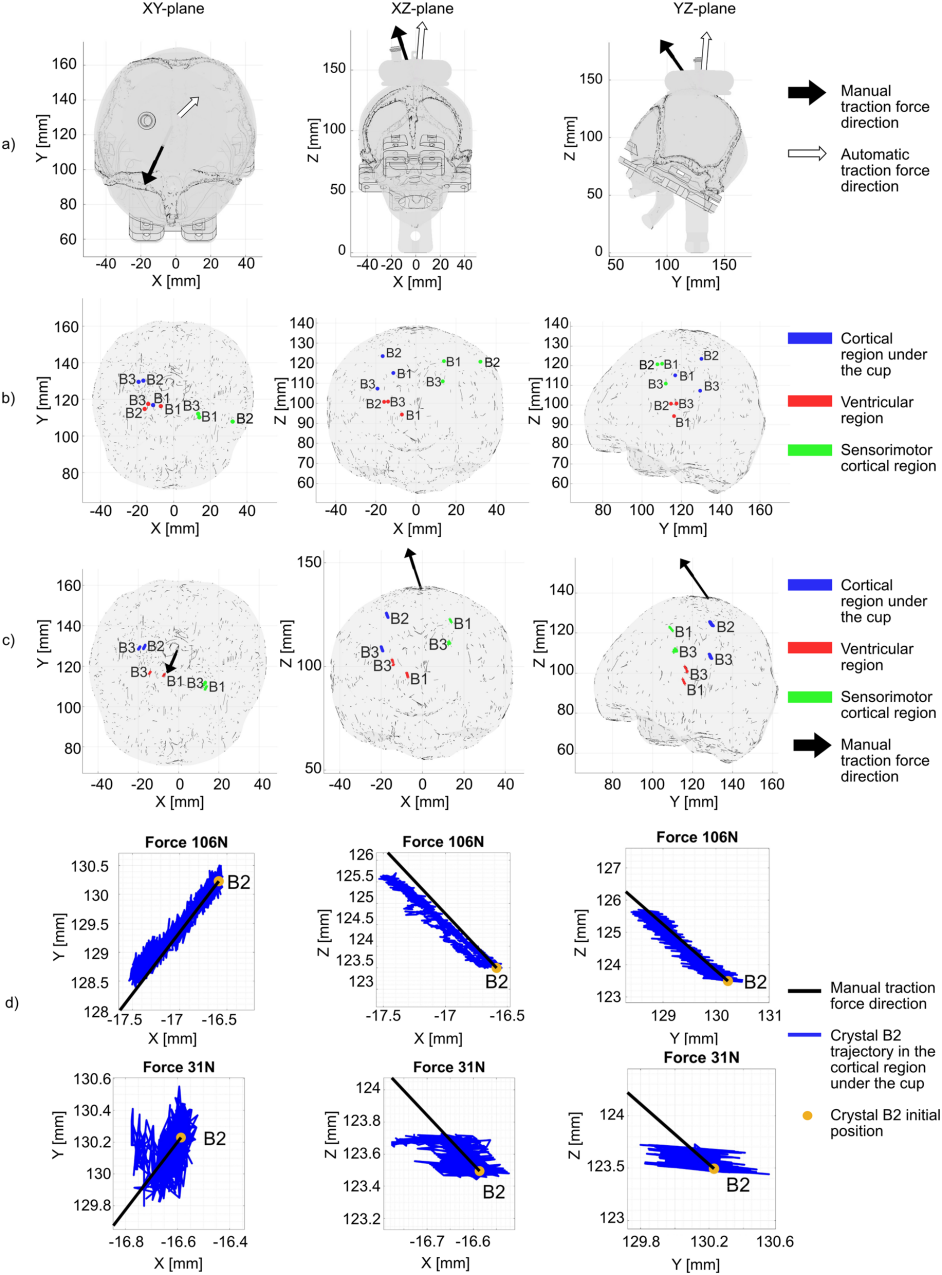
Schematic of the fetal head phantom, brain phantom with initial crystal positions and trajectories and directions of traction force in the initial reference frame. **a** Traction force direction in the manual and automatic force setup, **b** initial positions of the brain crystals (B1, B2, B3) for each crystal set (cortical region under the cup, ventricular region and sensorimotor cortical region) in the brain phantom (realistic scale of the markers with respect to the piezoelectric crystals), **c** trajectories of the selected crystal pairs at maximum manually applied traction force (cortical region under the cup: B2–B3, 106 N, ventricular region: B1–B3, 111 N, sensorimotor cortical region: B1–B3, 108 N) (realistic scale of the amplitude of the crystal trajectories with respect to the brain phantom), and **d** zoom on the trajectory (blue line) and initial position (yellow dot) of the brain crystal B2 in the cortical region under the cup at minimum (31 N) and maximum (106 N) manually applied traction force with traction force direction marked with the black line

In addition, small standard deviations across the three crystal sets in the manual force setup suggest that the mean direction of crystal trajectories could be considered as the direction of the applied traction force in this setup. The mean direction of crystal trajectories in the automatic force setup differed from the one in the manual force setup, resulting in different estimated directions of the traction force in the two setups (Fig. 5a).

Table 4 summarizes the distances between each pair of brain crystals for each crystal set, which was used to select one crystal pair for the strain computation. To isolate and capture the strain in the direction of the applied traction force, the crystal pair with best alignment in the direction of the traction force (i.e., small inter-crystal distances in directions perpendicular to the traction force direction) and large inter-crystal distances in the direction of the traction force were selected. Therefore, crystal pair B2–B3 in the cortical area under the cup and crystal pair B1–B3 in the ventricular region and in the sensorimotor cortical region were chosen as highlighted in Table 4.

**Table 4:** Inter-crystal distance for each crystal pair (B1–B2, B1–B3, and B2–B3) and for each crystal set (cortical region under the cup, ventricular region, and sensorimotor cortical region) with the selected crystal pairs for each crystal set highlighted in bold

Crystal set	Crystal pair	Inter-crystal distance (mm)			
		Force direction	First normal direction to the force	Second normal direction to the force	Inter-crystal direction
*Manual force setup*					
Cortical region under the cup	B1–B2	0.65	4.85	15.89	16.63
	B1–B3	11.15	11.80	6.25	16.86
	**B2–B3**	**11.80**	**6.14**	**9.65**	**16.43**
Ventricular region	B1–B2	8.15	6.91	2.57	10.99
	**B1–B3**	**6.22**	**5.33**	**4.86**	**9.52**
	B2–B3	1.93	1.57	2.29	3.39
Sensorimotor cortical region	B1–B2	3.65	17.79	2.61	18.35
	**B1–B3**	**8.89**	**3.13**	**4.15**	**10.30**
	B2–B3	5.23	20.92	1.53	21.62
*Automatic force setup*					
Cortical region under the cup	B1–B2	9.34	3.03	13.42	16.63
	B1–B3	7.01	3.27	14.98	16.86
	**B2–B3**	**16.36**	**0.23**	**1.56**	**16.43**

Figure 5 presents the schematics of the fetal head phantom, brain phantom with initial crystal positions, traction force direction, as well as crystal trajectories in the initial reference frame. Figure 5a displays the direction of the traction forces from Table 3 in both experimental VAD testing setups. The automatic traction force direction nearly aligns vertically with the neck bones, while the direction of the manual traction force is slightly oriented toward the front of the fetal head phantom. Figure 5b shows the schematic of the brain phantom with the initial brain crystal positions. The brain crystals are positioned in clusters for the different crystal sets. However, they are not precisely positioned in the intended regions, due to imprecision in the crystal placement method, such as crystal B2 of the sensorimotor cortical region positioned at the surface of the brain phantom and crystals B2 and B3 of the ventricular region located adjacent as also noted in Table 4.

Figure 5c shows the crystal trajectories from the selected crystal pair of each crystal set (Table 4) at the maximum manual traction force. Notably, all brain crystals, regardless of their location or set, move in the direction corresponding to the applied traction force as reported in Table 3. Figure 5d zooms on the trajectory of crystal B2 of the cortical region under the cup at maximum and minimum manual traction force. The crystal moves away from its initial position in the traction force direction and returns to its initial position, which is also illustrated in Fig. 5c. Small differences between the crystal trajectory and the direction of the traction force, especially in the *XZ* plane at a traction force of 106 N, are attributed to the variability in the crystal trajectory direction in the manual force setup (Table 3). Moreover, higher forces result in greater displacements in the direction of the traction force.

### Repeatability

**Fig. 6:**
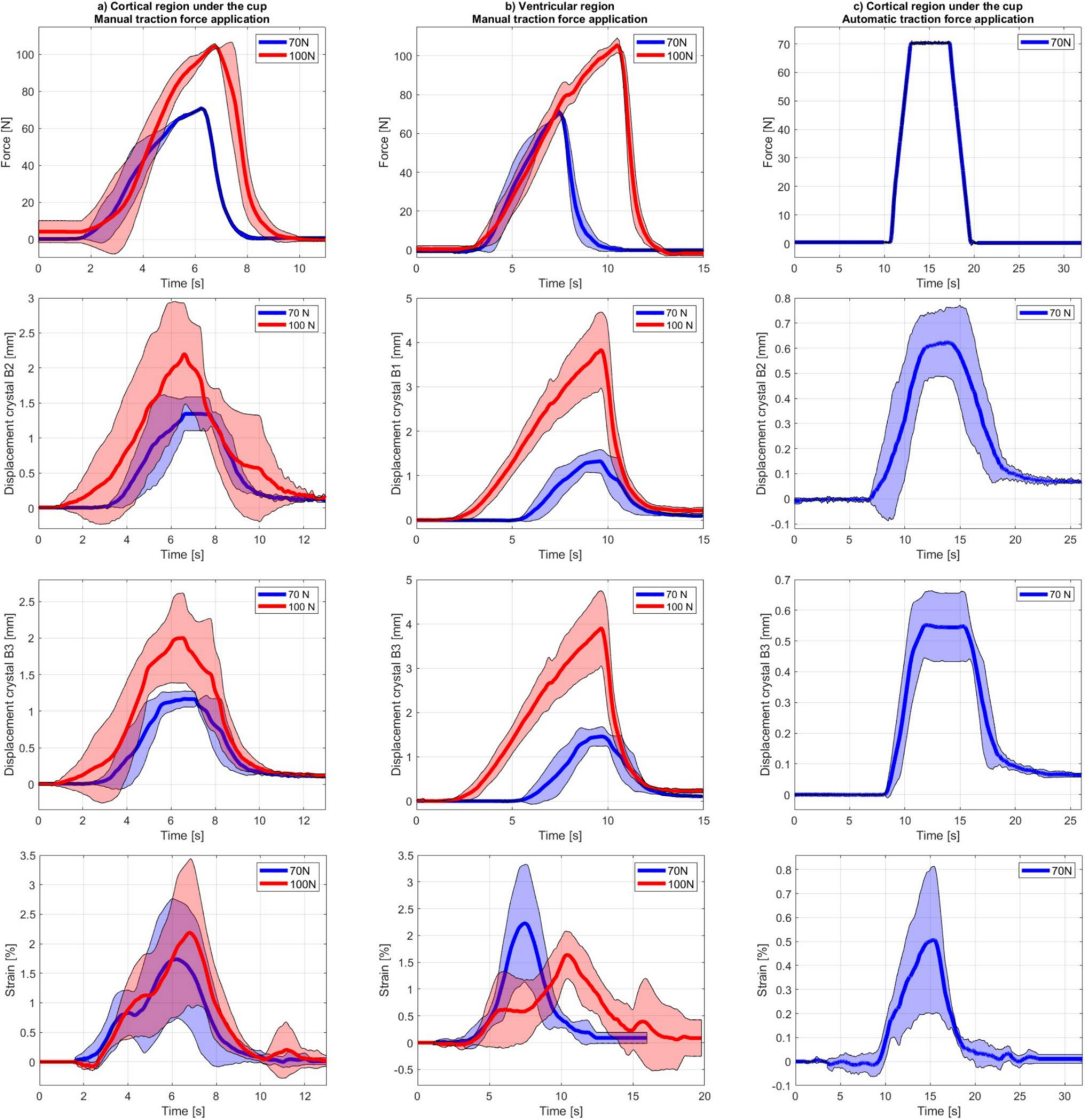
Traction force, crystal displacement, and corresponding strain over time in the direction of the traction force, over four repeated experiments, for **a** crystal pair B2–B3 of the cortical region under the cup in the manual force setup reaching a peak around 70 N and 100 N, **b** crystal pair B1–B3 of the ventricular region in the manual force setup reaching a peak around 70 N and 100 N, and **c** crystal pair B2–B3 of the cortical region under the cup in the automatic force setup reaching a peak around 70 N with the peaks of the traction forces and the strain amplitudes aligned in post-processing for each crystal set and experimental VAD testing setup. The colored straight line represents the mean and the colored area the standard deviation

The force, crystal displacement, and strain curves from the four repeated experiments are shown in Fig. 6, whereas Table 5 summarizes the peak force, crystal displacement, and strain from the repeatability tests. In the manual force setup, the force profiles of both crystal sets present high repeatability throughout the time of the experiment and at peak values, which indicates consistent application of the traction force. The strain and crystal displacement patterns, characterized by a single positive peak, remained consistent across all experiments, resulting in similar strain and crystal displacement profiles under repeated traction forces. However, the quantification of the positive strain peaks has low repeatability, as evidenced by the high standard deviation shown in Fig. 6, in contrast to the more consistent traction force measurements. Table 5 further illustrates this for both forces (70 N and 100 N) and crystal sets. When expressing the repeatability as standard deviation over the mean, it ranges between 26 and 61% for strain peaks, between 9 and 33% for crystal displacement peaks, whereas it is below 3.5% for the traction force peak. Higher crystal displacements and strain peaks are observed at 100 N than at 70 N, except for the ventricular region where higher strain and lower displacements are observed at 70 N than at 100 N. It can also be noted that the amplitudes of the crystal displacement are higher in the ventricular region compared to the cortical region under the cup.

**Table 5:** Force peaks, crystal displacement peaks, and strain peaks over four repeated experiments at traction forces around 70 N and 100 N for the selected crystal pair of each crystal set; crystal pair B2–B3 of the cortical region under the cup in the manual and automatic force setup, and crystal pair B1–B3 of the ventricular region in the manual force setup

	Force peak: mean (SD, N)		Crystal displacement peak: mean (SD, mm)				Strain peak: mean (SD, %)	
Traction force (N)	70	100	70	100	70	100	70	100
Crystal set								
*Manual force setup*								
			Crystal B2		Crystal B3		Crystal pair B2–B3	
Cortical region under the cup	70.83 (0.24)	104.49 (1.46)	1.35 (0.24)	2.20 (0.72)	1.17 (0.11)	2.00 (0.62)	1.74 (1.00)	2.19 (1.24)
			Crystal B1		Crystal B2		Crystal pair B1–B2	
Ventricular region	70.96 (1.73)	105.49 (3.76)	1.32 (0.26)	3.83 (0.86)	1.46 (0.22)	3.90 (0.85)	2.23 (1.10)	1.63 (0.44)
*Automatic force setup*								
			Crystal B2		Crystal B3		Crystal pair B2–B3	
Cortical region under the cup	70.71 (0.27)	–	0.63 (0.14)	–	0.55 (0.11)	–	0.51 (0.31)	–

In the automatic force setup, the force profiles present higher repeatability and the standard deviation at the force peak is slightly smaller than in the manual force setup. Similar to the manual force setup, the strain profiles in the automatic force setup exhibit lower repeatability compared to the crystal displacements and the force profiles. Despite similar relative standard deviation at strain peak, the strain amplitude is lower in the automatic force setup than in the manual force setup for the equivalent crystal set and traction force (cortical region under the cup, 70 N). The same observation holds for the crystal displacements.

In contrast to the low repeatability in strain measurements, the repeatability of inter-crystal peak distances measured before and after trilateration was high. The repeatability in inter-crystal peak distances measured via sonomicrometry, expressed as the standard deviation over the mean, ranged from 0.05 to 0.2%. Despite slightly higher, the repeatability of the inter-crystal peak distances computed using the 3D coordinates of the brain crystals after trilateration remained small, ranging from 0.16 to 0.49%.

### Strain vs. Force

**Fig. 7:**
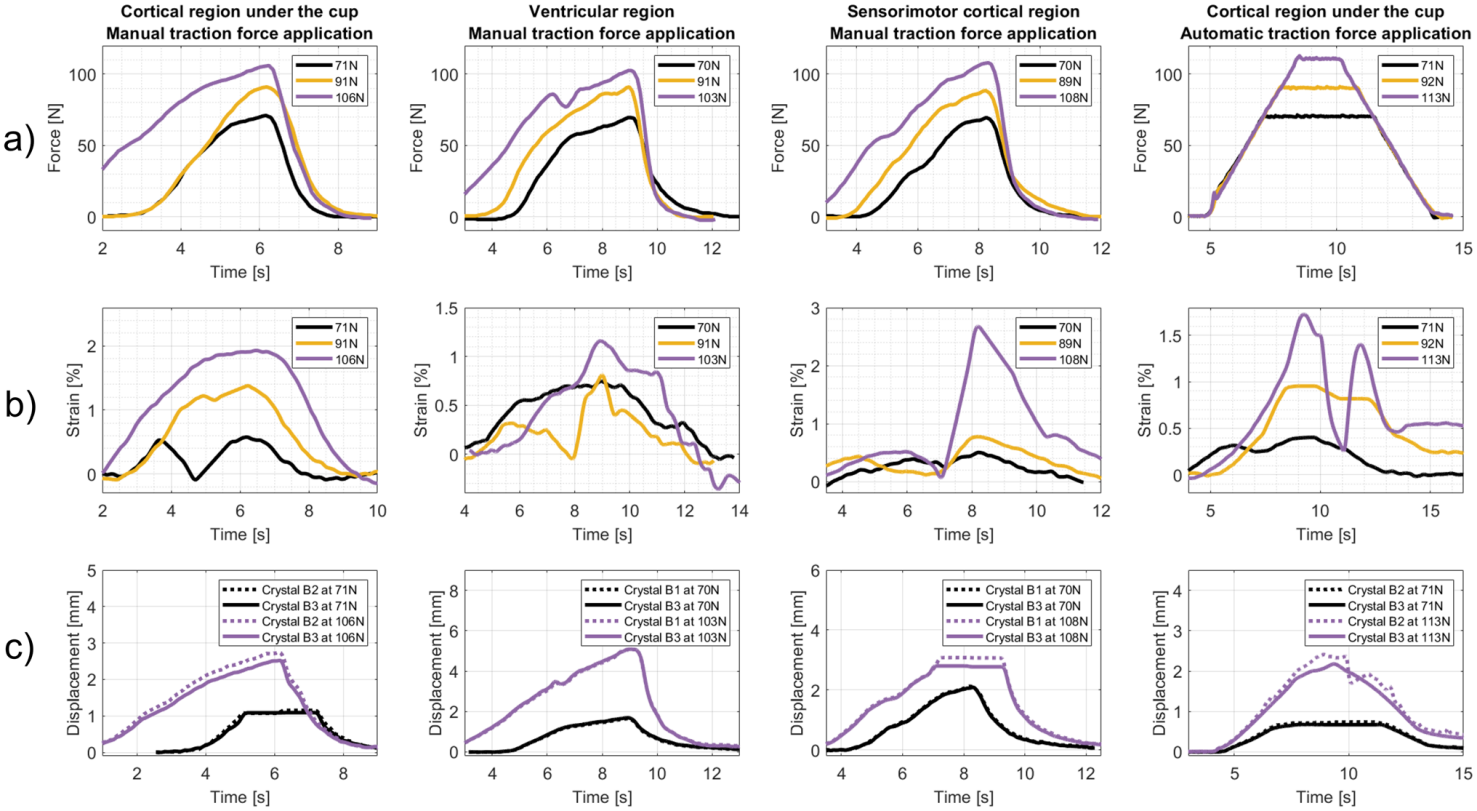
Sample traction force (**a**), strain (**b**), and crystal displacement curves (**c**) for crystal pair B2–B3 in the cortical region under the cup in the manual force setup, crystal pair B1–B3 in the ventricular region in the manual force setup, crystal pair B1–B3 in the sensorimotor cortical region in the manual force setup, and crystal pair B2–B3 in the cortical region under the cup in the automatic force setup

Sample traction force, strain, and crystal displacement profiles are shown in Fig. 7. Figure 7a presents the traction force curves and the reached traction force peaks (in the legend) for three sample experiments applying around 70 N, 90 N, and 100 N traction force for each crystal set in both experimental VAD testing setups. The strain curves vary with changes in applied traction force (Fig. 7b). Yet the strain profiles are generally less consistent than the traction force profiles, particularly in the ventricular region and sensorimotor cortical region. A distinct atypical strain profile in the cortical region under the cup is observed when applying 113 N in the automatic force setup, featuring a double peak typically seen for the last experiments when the fetal head phantom was slightly broken. Overall, higher strains are observed when higher traction forces are applied, except for the ventricular region in the manual force setup, where strain at 70 N exceeds the strain at 91 N during major parts of the time period of applied traction force. Regardless of the crystal set or experimental VAD testing setup, the applied traction force induces tension in the brain phantom in the direction of the traction force, evidenced by positive strain amplitudes. The strain amplitude is lower in the ventricular region (average strain peak of 1.63% at around 100 N) compared to the other two regions in the manual force setup (average strain peak of 2.20%/2.68% at around 100 N/108 N in the cortical region under the cup/sensorimotor cortical region). In the automatic force setup, the strain is lower compared to the equivalent experiments in the manual force setup.

Figure 7c presents samples of displacement curves under traction forces of approximately 70 N and 100 N in both experimental VAD testing setups. Similarly to strain profiles, displacements vary with traction forces in all experiments and reach an average of 2.21 mm, 3.90 mm, and 3.10 mm at a traction force of approximately 100 N, respectively, in the cortical region under the cup, the ventricular region, and the sensorimotor cortical region. In contrast to strain, greater crystal displacements occur in the ventricular and sensorimotor cortical regions than in the cortical region under the cup. The crystal displacements differ more between the two crystals at 100 N than at 70 N, which results in higher strain at 100 N than at 70 N. This is observed in all experiments except in the ventricular region at 103 N, where the strains are smaller than for the other crystal sets. For each crystal set, both crystals in the pair exhibit positive displacements.

**Fig. 8:**
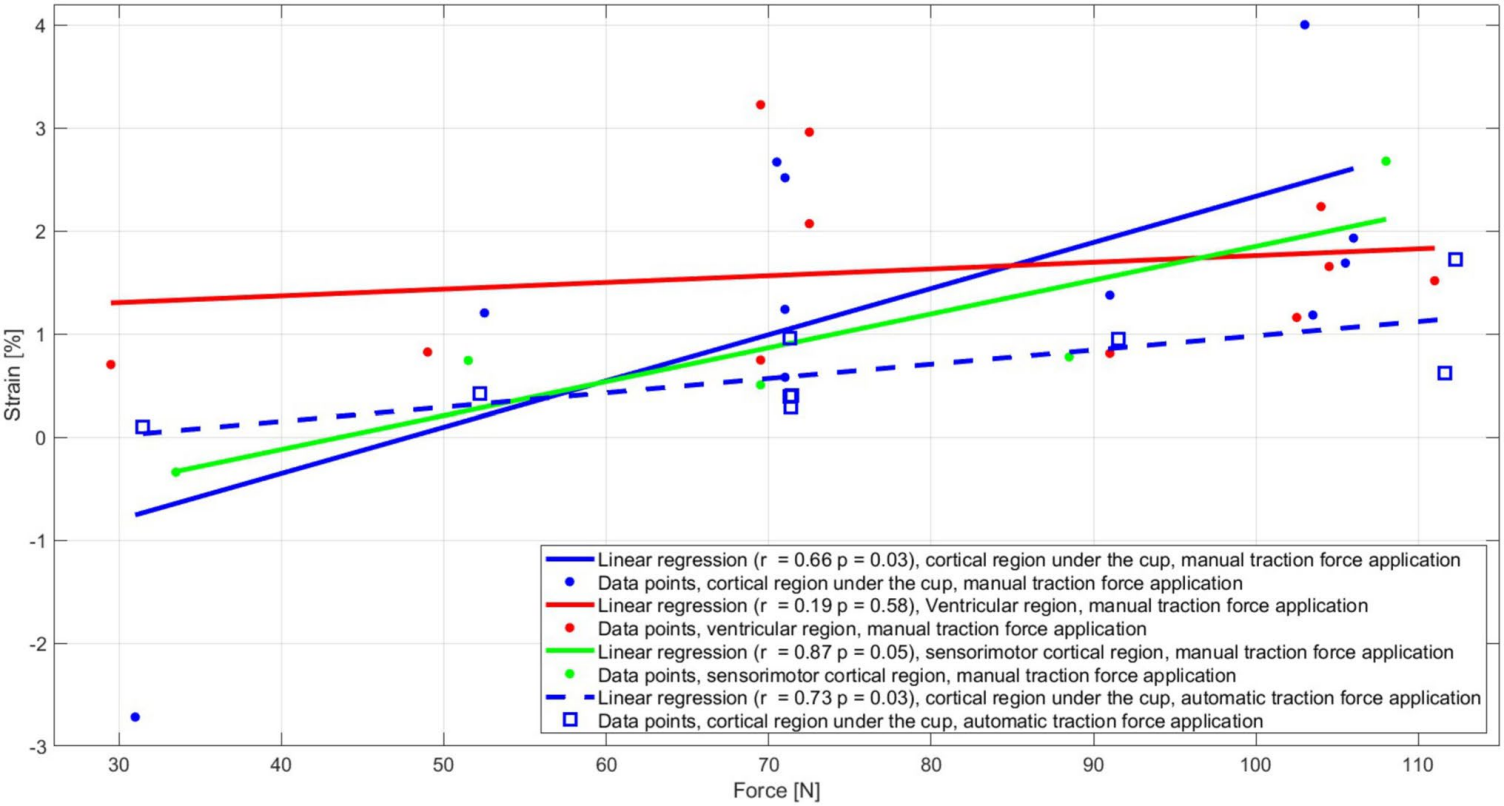
Correlation between the force and strain peaks of each crystal set in both experimental VAD testing setups where the dots represent estimated strain and force peaks, the full line is the linear regression with the associated *r* and *p* values in the manual force setup (blue: cortical region under the cup, red: ventricular region, green: sensorimotor cortical region), the squared markers represent the strain and force peaks, and the dashed line is the linear regression in the automatic force setup with the associated *r* and *p* values (blue: cortical region under the cup)

Figure 8 shows the data points of the computed strain peaks and associated traction force peaks as well as the correlation between force and strain peaks for the different crystal sets and experimental VAD testing setups. In the manual force setup, statistically significant correlations were found in the cortical region under the cup (*r* = 0.66, *p* = 0.03), and in the sensorimotor cortical region (*r* = 0.87, *p* = 0.05), while no correlation was found in the ventricular region (*r* = 0.19, *p* = 0.58). In the automatic force setup, a statistically significant correlation was found in the only tested set (cortical region under the cup) (*r* = 0.73, *p* = 0.03). Strain amplitude ranges between 0 and 4%, with the highest strains in the cortical region under the cup in the manual force setup. Some outliers can be observed such as high strains at 70 N in the ventricular region and negative strains at 30 N in the sensorimotor region and in the cortical region under the cup in the manual force setup.

## Discussion

In this study, brain strain was quantified in different brain regions in 39 experiments on the fetal head phantom by applying traction forces up to 112 N, which shows the feasibility of the experimental VAD testing setup to investigate the strain–force correlation. To the best of our knowledge, this study is the first to quantify brain strain caused by traction forces during experimental VAD and to show a correlation between brain strain and traction force. Positive strain was observed in most cases regardless of brain region and traction force amplitude (Fig. 7), with higher crystal displacement close to the force application point, resulting in brain stretching in the direction of the applied force.

### Crystal Displacement and Strain Quantification

When applying approximately 100 N of traction force, the brain experienced a maximum strain of 4% and an average crystal displacement of approximately 3 mm (Figs. 7, 8). Since deformation in the fetal brain during delivery is an unexplored field, comparative studies are difficult to find. When comparing with findings from in silico and PMHS studies on impact brain injury, the strains observed in our study are relatively low. For instance, Alshareef et al. [54] measured an average crystal displacement of 5 mm (up to 14 mm) in PMHS adult brains under injurious rotational loading conditions. Similarly, Hardy et al. [37, 55] reported total brain movement of around 5 mm and maximum principal strain averaging 8.8%. Zhou at al. studied traumatic brain injury in PMHS due to linear acceleration and found principal strain ranging from 7 to 22% [38]. In silico studies, such as Svein et al., showed varying maximal strains depending on the loading, ranging from 3 to 10% of maximum principal strain [56]. Limited research examines traumatic brain injury in infants at 1.5 years of age, revealing higher strain levels compared to older children. For instance, studies show Green–Lagrange strains exceeding 15% and up to 30%, even when wearing a bicycle helmet [57], and first principal brain strain reaching 55% with an average of 36% from playground falls [58]. However, the literature primarily examines traumatic brain injury from injury-inducing movements in automotive accidents and sports, unlike the quasi-static VAD traction force in this study. In addition, most studies focus on adults or infants over 1.5 years of age, whose brain strains are not comparable to those of fetuses because infant brain strain varies with age due to unique anatomical and biochemical properties [57]. Methodological differences, including in silico or PMHS settings and various types of strain, also complicate comparisons.

Some in vivo studies, examining strain and crystal displacement during non-injurious head movements by adult volunteers, found comparable levels of strain and displacement than those observed in this study, suggesting that the levels observed in this study do not cause harm [59, 60]. This suggestion is also confirmed by the lower strain and displacement obtained in this study compared to literature describing traumatic brain injury [37, 38, 54, 58] and other studies assessing risk of adult brain injury from head impacts [56, 61–63]. This result aligns with clinical observations of low risk of neonatal morbidity (short/long-term fetal complications) with low traction forces applied during VAD [21, 23]. However, the strain computation in this study is limited to the direction of the traction force, which may underestimate the overall strain in a 3D biomechanical context. Although the direction of the traction force is assumed to capture the majority of the biomechanics of the brain deformation caused by the traction force (Fig. 5c, d; Table 3), this approach does not account for potential strain contributions from other directions. In addition, studying fetal brain injury at higher traction forces and the impact of the duration of traction force, even at low amplitudes is crucial as suggested by clinical observations [26]. Furthermore, injury mechanisms beyond brain deformation are believed to contribute to neonatal VAD complications [15, 17, 18, 21, 22, 25]. These may include hematomas and bleeding resulting from scalp and subcutaneous tissue injuries caused by the suction cup (particularly cup detachment and cup misplacement), as well as reduced brain oxygenation due to altered blood perfusion within the fetal head. While these mechanisms were outside the scope of this study, they present valuable areas for future research.

### Biomechanics of Brain Phantom Deformation

All crystal displacements in the main direction of motion (traction force direction) present an increase in amplitude with increasing traction force, regardless of the brain region (Figs. 6, 7c). In addition, higher crystal displacement but lower strain amplitude at high traction forces was observed in the ventricular region compared to the cortical regions (Figs. 6, 7; Table 5). This could be due to cortical regions near the suction cup being constrained by the rigid skull phantom, limiting movement of the brain phantom material, while the ventricular region, being less restricted, experienced more rigid body motion with minimal internal deformation, resulting in lower strain. However, the amplitudes of the crystal displacements in the ventricular region exceed 30% of the initial distances between crystals (Tables 4, 5), questioning the use of small strain theory [64]. For the other crystal sets, strain computations were based on crystal displacements below this threshold involving minimal geometric change [64, 65]. The large displacements in the ventricular region, suspected to be due to rigid movement, require nonlinear strain models, significantly increasing the complexity and risk of error in the strain computation [64, 65].

The strain amplitude tended to be higher in the cortical regions compared to the ventricular region in the manual force setup at high traction forces (Figs. 7b, 8). Furthermore, the correlation between brain strain and traction force in the cortical regions, but not in the ventricular region (Fig. 8), may confirm that distal brain regions close to the suction cup are more susceptible to high strain amplitudes at high forces than proximal brain regions. This could potentially lead to negative effects on the brain as suggested by studies in traumatic brain injury showing that higher brain strain amplitudes may increase the risk of injury [56]. However, strain quantification in the ventricular region was not accurate due to large crystal displacement in relation to inter-crystal distance (Tables 4, 5). In addition, the negative strain at low traction force was likely caused by minimal crystal displacement, which resulted in inaccurate strain computation in the traction force direction. Furthermore, those quantitative results need to be taken with precaution due to variability in estimated strain values over repeated measurements, low strain amplitudes, and limitations of the strain computation method that only captures 1D strain in the applied traction force direction. As such, lower strain amplitude is expected due to the projection of a brain deformation in 3D to one direction and the dependency of the crystal positions relative to each other as well as the dependency of the crystal positions relative to the direction and application point of the traction force.

In the automatic force setup, lower brain strain and unexpected strain patterns (Fig. 7b) compared to similar conditions in the manual force setup may be attributed to the weakened fetal head phantom, which had undergone 30 prior experiments in the manual force setup. The potential weakening of the fetal head phantom during the automatic force setup complicates direct comparisons between the two VAD testing setups. Consequently, the findings from the automatic force setup highlight its potential and feasibility rather than providing quantitative results.

### Experimental VAD Testing Setup

The high precision and accuracy of the estimation of crystal position (Table 2) and the constant initial position of each crystal after each experiment prove that the brain phantom was not experiencing plastic damage (Table 2; Figs. 5d, 7c). The method of combining sonomicrometry and trilateration, validated by Alshareef et al. to compute brain strain [54, 66, 67], allowed the computation of brain strain from the observation of the motion of the brain crystals. The brain crystals moved in a common direction regardless of the crystal set or amplitude of traction force (Table 3; Fig. 5c). In addition, all displacements were positive and varied with changes in the traction force (Fig. 7c), confirming that crystals moved in the force direction before returning (Fig. 5d), which validated the definition of the traction force direction, and the measurement of crystal displacement induced by the traction force.

The two experimental VAD testing setups in this study present different traction force directions due to the inherent differences between these two setups which have distinct advantages and limitations. The automatic setup allowed for examining strain variability independently of human factors in the application of traction force (amplitude, profile, and direction) for repeated measurements. Future studies could explore real clinical scenarios by applying traction force curves collected during in vivo studies [26]. On the opposite, the manual setup has potential for simulating diverse delivery scenarios and training obstetricians to minimize brain strain, and optimize VAD safety. Moreover, the profiles of the manual traction force exhibited high repeatability, whereas the strain profiles showed significantly lower repeatability compared to the force profiles in both experimental setups (Fig. 6), suggesting that variability in strain amplitude in the manual force setup is not attributable to human inconsistencies in applying the traction force. However, this deduction may be challenged by the use of a damaged fetal head phantom that may have caused variability in strain profiles in the automatic force setup.

In both setups, despite similar strain profiles observed for repeated traction forces (Fig. 6), low repeatability (high standard deviations over the mean) emphasizes the importance of conducting numerous experiments. Extending the strain computation method to 3D strain quantification may also enhance the repeatability in strain, as the higher repeatability of crystal displacements compared to the strain repeatability (Table 5) suggests that calculating 1D strain significantly impacts repeatability, and consequently, the accuracy of strain quantification. The higher repeatability of the inter-crystal distances compared to the crystal displacements also shows that while the measurement and post-processing of inter-crystal distances are reliable, it struggles to accurately quantify the small crystal movements induced by the traction force, contributing to the low repeatability in the strain peaks. The high repeatability of the inter-crystal peak distances (standard deviation below 0.5% of the mean), whether measured by sonomicrometry or calculated via trilateration, suggests that the low repeatability in strain (standard deviation ranging between 26 and 61% of the mean) is not attributable to poor repeatability in the sonomicrometry measurements or the trilateration algorithm. This also demonstrates the reliability of the trilateration, i.e., it is able to accurately reproduce the inter-crystal distances measured by sonomicrometry at high repeatability.

The developed skull phantom was previously validated by Jones et al. [41] and Khalid [42] who implemented a fetal skull phantom with materials that best represent the mechanical properties of its components as reported in Table 1, despite not considering the orthotropic properties of fetal skull. Designing a skull phantom is challenging due to the large variability in the fetal skull properties and the limited availability of suitable 3D-printing materials. McPherson and Kriewall [50] reports thicknesses of the skull bones of full-term fetuses ranging between 0.71 and 0.86 mm. These values are smaller than our adopted value of ~ 1.6 mm that is based on studies by Roth et al. [68] (1.4 mm in a 17-day-old infant), Coats et al. [69] (1.2 mm in a 5-week-old infant) and segmentation from our CT datasets. This value is justifiable considering that we assess VAD for larger or post-term fetuses [1]. It should also be noted that smaller values are close to the lower threshold of our Polyjet 3D-printing technology, which would increase the risk of fracture and weaken the interfaces between skull bones and sutures/fontanelles. In future work, we aim to investigate alternative printing technologies so that we can also create term fetuses with thinner skull bones. The screwing system, designed to preserve the integrity of the fetal head, introduced force concentration at the bolted joints, affecting stress distribution. Optimizing screw placement and separation aimed to minimize this impact, particularly in the upper skull phantom (red in Fig. 1a), which is considered key to brain deformation under traction force from the suction cup.

The CH developed by Forte et al. [53] was selected as the brain phantom material in this study due to its close replication of human brain tissue, especially under low to medium strain rates, suitable for simulating brain loading during VAD. While most brain model materials used in the literature, like gelatin and PVA, are suited for traumatic brain injury studies [42, 70–72], they fail to replicate the nonlinear, hyperelastic, and viscoelastic behavior of the brain as effectively as the CH of Forte et al.. However, the CH material mimics properties of the adult brain, not newborns’ brain. In addition, CH models are used in surgical training, but not for the specific context of vaginal delivery. Furthermore, the density and Poisson's ratio of CH have not been definitively correlated with the mechanical properties of a newborn's brain. Despite these drawbacks, key attributes of CH justify its use in this study. In addition, the brain phantom did not mimic the complex geometry of human brain and lacked important brain structures, such as lateral ventricles, which could affect the distribution of the investigated brain strain. This study also did not model the meninges, thus, omitting the complex skull–brain interface, similar to some numerical studies of vaginal delivery [48, 73]. While incorporating these details would be valuable, the complexity of modeling these structures in an experimental setup is beyond the scope of this study. The cuts in the brain phantom, made for easier insertion into the small opening of the upper part of the skull phantom, were small and located in regions considered to have limited implications in this simplified brain phantom. While it could have affected the computed brain strain, we consider its effect minor as the reinserted pieces remain secure due to the brain’s large volume in the skull cavity, preserving overall integrity.

A major limitation of the fetal head phantom was the lack of a scalp model, which prevents the study of suction cup and scalp interaction, likely important for common short-term neonatal complications [4, 16], and could also affect the measured brain strain. Investigating whether this interaction allows for higher traction forces than the vacuum force of the suction cup (*F* = pressure * contact area) would also be valuable. The mechanics of cerebrospinal fluid within the skull is not accounted for, in addition to the fact that the fetal head phantom is not fully immersed, potentially affecting brain movement. Sonomicrometry wires within the brain phantom could also influence the movement of the brain during the experiments.

Moreover, the experimental VAD testing setup did not replicate forces from the birth canal during delivery, nor the orientation of the fetal head in the birth canal. The fragility of the fetal head phantom in the experimental VAD setup and cup detachment at traction forces above 110 N limited the number of experiments and the maximum traction force that could be applied. Moreover, the application of traction force did not mimic the traction force amplitude, direction, and pattern during repeated uterine contractions. Nonetheless, this study focused on measuring the impact of the amplitude of VAD traction force on the fetal brain by mimicking the onset phase of the typical traction force curve used in clinical settings. Additional improvements may include monitoring force direction and synchronizing measurement systems (sonomicrometry and force gage).

Another limitation of this study is the limited number of active channels of the sonomicrometry system used in this study, allowing measurements of only three brain crystals at a time. Consequently, only 1D strain computation was feasible, relying on the relative displacement between two crystals. Also suggested by Alshareef et al. [54], increasing the number of crystal displacement measurements would enable 3D strain computation which is more robust, facilitate comparison with FEM results, and enable exploration of more refined regions with the same number of experiments. Distinct differences in biomechanical response observed between the cortical and ventricular regions under VAD traction forces highlight the limitations of the 1D strain computation used in this study. The significant strain–force correlation in the cortical region under the cup in the traction force direction and observed compressive strain in both normal directions to the traction force (data not shown), suggests that localized 1D analysis may be sufficient to capture brain deformation in this region. However, the absence of such correlation in the ventricular region in the traction force direction, and observed compressive and tensile strain in each of the normal directions to the traction force, underscores the need for 3D strain computation. This would be needed to fully understand the multidirectional strain pattern in brain regions further from the source of traction force that cannot be captured when computing strain only in the traction force direction. Besides the sensitivity of the computation of 1D strain to crystal position, inadequate control of crystal positioning within the brain mold resulted in broad regions for strain computation.

Despite the limitations of the study, which pose challenges for interpretation and quantification, the fetal head phantom and experimental VAD setup provided valuable preliminary insights into brain deformation caused by traction forces during VAD.

### Future Perspectives

Commonly, biomechanical investigations employ FEM for detailed brain responses to various loads. Yet, FEM models simulate complex systems based on assumptions (e.g., material properties, boundary conditions, shape functions, mesh size, and mesh type), and a major challenge is the need for experimental or in situ data to ensure the results of the model to reflect real-world behavior. The results from this study could serve as a baseline for validating FEM models of VAD as well as input data to build numerical simulations of VAD. This has been emphasized in similar studies using sonomicrometry or markers to measure tissue deformation [37, 54, 74]. This setup has significant potential for development into a numerical model, enabling further investigation beyond the physical constraints of the current configuration (such as valuable numerical studies on vaginal delivery and VAD [22, 24, 48]).

Besides numerical studies, enhancing the experimental VAD testing setup and fetal head phantom, as discussed in this study—such as incorporating a scalp model—could facilitate the exploration of various clinical scenarios. These include variations in traction force amplitudes and directions, different cup placements, detachment events, and suction cup characteristics (e.g., manufacturer, material, and dimensions), all of which are critical factors. The experimental VAD testing setup could allow bridging the gap between the virtual world (FEM model) and the physical world (VAD device) to enhance understanding of VAD biomechanics and establish biomechanical evidence for safe VAD including guidelines for device and procedural optimization to reduce failures and neonatal complications. Future research can benefit from both FEM and experimental setups to study brain deformation in VAD as the experimental setup gives direct biomechanical insights through mechanical response measurements, while FEM offers detailed modeling. With limited research on fetal brain deformation in VAD, both methods, despite their challenges, are essential for advancing knowledge in this underexplored field.

In conclusion, this study developed a fetal head phantom for VAD testing in manual and automatic setups with forces up to 112 N. Brain deformation was measured using sonomicrometry, showing significant correlations with traction force, especially near the suction cup. Despite limitations such as the lack of scalp modeling and limited 1D strain computation, the setups demonstrated feasibility and potential for future work on safe VAD guidelines and training platforms.
